# Diagnostic Criteria for the Painful Swollen Pediatric Knee: Distinguishing Septic Arthritis From Aseptic Effusion in a Non-Lyme Endemic Area

**DOI:** 10.3389/fsurg.2021.740285

**Published:** 2021-11-01

**Authors:** Claudia S. Thomas, Corey J. Schiffman, Anna Faino, Viviana Bompadre, Gregory A. Schmale

**Affiliations:** ^1^Department of Orthopaedics and Sports Medicine, University of Washington, Seattle, WA, United States; ^2^Seattle Children's Hospital, Seattle, WA, United States; ^3^Department of Orthopedics and Sports Medicine, Seattle Children's Hospital, Seattle, WA, United States

**Keywords:** musculoskeletal infection, septic knee, knee effusion, swollen knee, pediatric septic arthritis

## Abstract

**Purpose:** The child with a painful swollen knee must be worked-up for possible septic arthritis; the classic clinical prediction algorithms for septic arthritis of the hip may not be the best models to apply to the knee.

**Materials and methods:** This was a retrospective case-control study of 17 years of children presenting to one hospital with a chief complaint of a painful swollen knee, to evaluate the appropriateness of applying a previously described clinical practice algorithm for the hip in differentiating between the septic and aseptic causes of the painful knee effusions. The diagnoses of true septic arthritis, presumed septic arthritis, and aseptic effusion were established, based upon the cultures of synovial fluid, blood cultures, synovial cell counts, and clinical course. Using a logistic regression model, the disease status was regressed on both the demographic and clinical variables.

**Results:** In the study, 122 patients were included: 51 with true septic arthritis, 37 with presumed septic arthritis, and 34 with aseptic knee effusion. After applying a backward elimination, age <5 years and C-reactive protein (CRP) >2.0 mg/dl remained in the model, and predicted probabilities of having septic knee arthritis ranged from 15% for the lowest risk to 95% for the highest risk. Adding a knee aspiration including percent polymorphonucleocytes (%PMN) substantially improved the overall model performance, lowering the lowest risk to 11% while raising the highest risk to 96%.

**Conclusions:** This predictive model suggests that the likelihood of pediatric septic arthritis of the knee is >90% when both “age <5 years” and “CRP > 2.0 mg/dl” are present in a child with a painful swollen knee, though, in the absence of these factors, the risk of septic arthritis remains over 15%. Aspiration of the knee for those patients would be the best next step.

## Introduction

A painful swollen knee is a common diagnostic dilemma for the providers treating children in an emergency room, urgent care, primary care, or specialty setting. The differential diagnosis should include fracture, ligamentous or soft tissue injury, osteochondral pathology, infection in or around the joint, hematologic effusions, rheumatologic processes, and neoplasm (1). Of these potential diagnoses, septic arthritis is a surgical urgency, requiring prompt diagnosis and treatment to avoid permanent damage in the form of chondrolysis, epiphyseal damage, ankylosis, and growth disturbance (2, 3). Currently, there are reliable clinical predictors for determining the presence of septic arthritis in the hip in children, previously described by Kocher and modified by Caird (4–6). These criteria include elevated serum white blood cell count (WBC) above 12,000 cells/mm^3^, erythrocyte sedimentation rate (ESR) above 40 mm/h, temperature above 38.5°C, non-weight bearing status, and C-reactive protein (CRP) level above 2.0 mg/dl. These same criteria are not verified for joints other than the hip, and no other algorithm is described to rule out septic arthritis in the knee.

### Rationale

Children with septic knee arthritis have been previously shown to follow Kocher's criteria less reliably than children with septic hip arthritis and tend to be younger than patients presenting with septic hip arthritis (4, 7, 8). In addition, a recent study showed that the presence of three or more positive criteria in the knee is only 49% sensitive for septic arthritis (9), compared with 84% sensitivity in the hip in Kocher's second validation study (5).

This study aimed to examine whether children with septic arthritis of the knee may be distinguished from the non-infectious causes of a painful knee effusion using the same predictive variables as described for the hip, as well as to suggest a new clinical prediction algorithm for distinguishing the septic knee arthritis from aseptic knee effusion, incorporating the data gained during the work-up from a knee joint aspiration (10).

## Materials and Methods

This was a retrospective case-control study of patients who presented at a tertiary care children's hospital between 2001 and 2017 in a non-Lyme endemic area. The potential patients for inclusion were identified in a search of the medical records for inpatient notes or emergency department notes with the terms “septic knee arthritis,” “septic arthritis of the knee,” ICD-9 code 711.06 (pyogenic arthritis and lower leg), or ICD-10 codes M00.861, 862, and 869 (arthritis due to other bacteria and knee), and who underwent knee joint aspiration—CPT code 20610 (aspiration of a large joint). The inclusion criteria for the study were age <18 years; a presenting complaint of knee pain; laboratory tests drawn including ESR, CRP, and complete blood count; aspiration of the knee with a culture of the aspirate, and orthopedic consultation to evaluate for septic knee arthritis. The exclusion criteria for the study were the absence of a synovial fluid aspirate or culture of the aspirate, diagnoses related to post-operative infections (e.g., after knee arthroscopy), hip transient synovitis, septic hip arthritis, ankle septic arthritis, or multiple septic joints.

The following data were collected for each patient: age at presentation, sex, chief complaint (pain, effusion, or unwillingness to ambulate), serum WBC count, ESR, CRP, weight bearing status, presence of knee effusion, peak temperature at presentation, range of motion arc, presence of surrounding erythema, synovial cell count, percentage polymorphonucleocytes (%PMN), synovial fluid culture results, blood culture results, and final diagnosis if determined. The subjective data points, such as the presence of knee effusion, range of motion arc, and presence of erythema, were gathered from the review of the orthopedic consultation note.

The aspirations were typically performed in our Emergency Department under conscious sedation. Arthrotomy of the knee was typically performed for the cloudy knee aspirates and when the patients presented with multiple signs and symptoms consistent with septic arthritis of the knee who failed to improve with observation.

**Figure 1:**
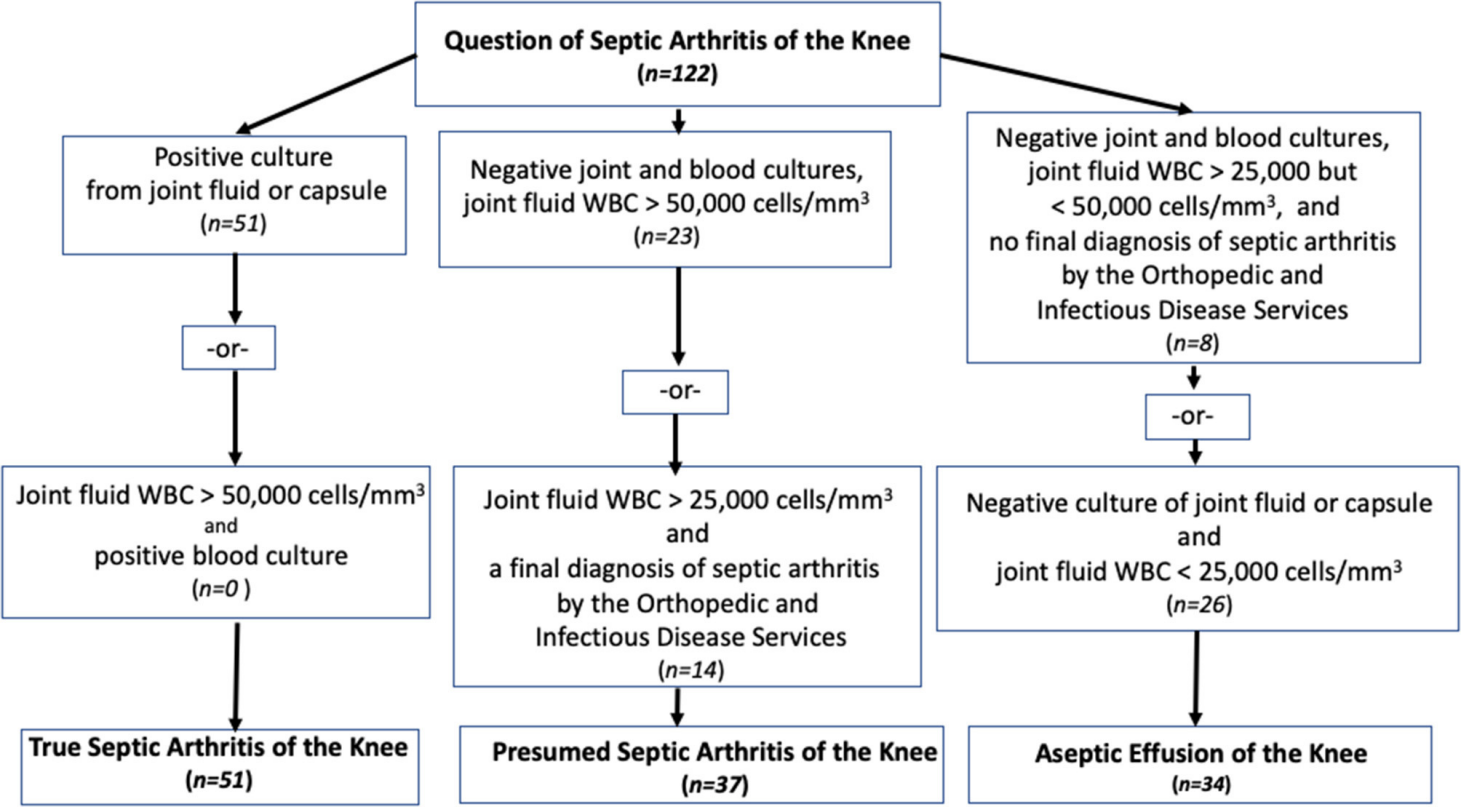
Algorithm for determining the patient groups.

The patients were categorized and explicitly assigned into three groups similar to Kocher's original study (4) with the addition of the modification described by Heyworth (11) and Obey (9) (Figure 1). The diagnosis of true septic knee arthritis was assigned to the patients with a positive culture of joint aspirate, or a joint aspirate WBC count >50,000 cells/mm^3^ with positive blood cultures. The diagnosis of presumed septic arthritis was assigned to patients with negative joint fluid and blood cultures, but with a joint fluid WBC count >50,000 cells/mm^3^, or a joint fluid WBC count >25,000 cells/mm^3^, and a final diagnosis of septic arthritis of the knee by the Orthopedic and Infectious Disease Services. These two groups were combined into one septic arthritis group. The diagnosis of aseptic knee effusion was assigned to the patients with a joint fluid WBC count between 25,000 and 50,000 cells/mm^3^, negative joint and blood cultures, and no final diagnosis of septic arthritis of the knee by both the Orthopedic and Infectious Disease services or, a cell count of <25,000 cells/mm^3^ and negative culture of the joint aspirate (Figure 1).

### Statistical Methods

Descriptive statistics were used to summarize the differences between a septic knee (true and presumed) and an aseptic knee, as well as the differences between the true and presumed septic knee. The *P*-values for comparisons were from Wilcoxon's rank sum test for the continuous variables and from Fisher's exact test for the categorical variables. Histograms were used to aid in the selection of cut points of interest for age, ESR, CRP, temperature, serum WBC count, and %PMN. Logistic regression models were then used to regress the disease status (septic knee vs. aseptic knee) on the predictor variables of interest. The patients with missing data were excluded from the logistic regression modeling. The model coefficients were summarized as odds ratios (*OR*s) and 95% *CI*. A backward elimination was performed on a model with *a priori* specified variables using a *p*-value cutoff of 0.15. The receiver-operator curves (ROC) and the areas under curves (AUCs) were calculated to assess the model performances, though the model performance measures were subjected to overfitting. DeLong's test was used for assessing the statistically significant differences in the model AUCs. The predicted probabilities were calculated for all the predictor variable combinations. A two-sided test *p*-value <0.05 was considered significant, and all the analyses were done using R version 3.5.1 (R-Core Team, Austria) (12).

### Compliance With the Ethical Standards

The authors report neither potential nor current conflicts of interest. No outside funds were received by any of the authors for the completion of this study. This study was approved by the Institutional Review Board (IRB) and informed consent was not required given the retrospective nature of the study.

## Results

A review of 464 charts identified 122 patients who met the study inclusion criteria. In using the diagnostic criteria described above, there were a total of 51 patients with “true septic arthritis,” 37 patients with “presumed septic arthritis,” and 34 patients with “aseptic knee effusion.” In this study, 342 patients were excluded for one of the criteria mentioned above, most commonly for diagnoses unrelated to the knee. In addition, 5 patients with missing data were excluded from the logistic regression models and additional 24 patients lacked the aspirate differentials and were excluded from the modeling which included %PMN. Univariable analysis comparing patients with septic knee arthritis to those with aseptic effusion revealed differences in presenting CRP, age, weight bearing status, as well as aspirate cell count and aspirate %PMN (Table 1).

**Table 1:** Comparison of septic knee vs. aseptic knee, continuous variables.

**Variable**	**Septic knee arthritis** ***n* = 88**	**Aseptic knee effusion** ***n* = 34**	***P*** **-value**
AGE, continuous	3.25 [1.47, 7.44]	8.46 [6.03, 13.82]	<0.001
Age < 5 years	57 (65)	6 (18)	<0.001
WBC, continuous	12.35 [10, 16.8]	11.35 [7.53, 15.1]	0.0851
WBC > 12k	49 (56)	15 (44)	0.3131
ESR, continuous	44 [26, 69]	36 [17.25, 56.75]	0.0842
ESR > 40	48 (56)	15 (44)	0.2316
CRP, continuous	4.5 [3.2, 7.53]	3.15 [1.22, 5.42]	0.0076
CRP > 2	78 (89)	22 (65)	0.0036
Temperature, continuous	38 [37.2, 38.68]	37.45 [37, 38.4]	0.2809
Temperature > 38.5	29 (34)	8 (24)	0.3806
Cell count^∧^	79,050 [47,342.5, 127,117.5]	16,241.5 [6673.75, 37,485]	<0.001
%PMN^∧^	0.9 [0.87, 0.94]	0.78 [0.65, 0.88]	<0.001
Male sex	53 (60)	24 (71)	0.3056
Positive effusion	78 (90)	30 (88)	0.756
Non-weight bearing	66 (75)	16 (47)	0.0049
Weight-bearing	16 (18)	15 (44)	0.0051
Positive erythema^∧^	52 (74)	20 (74)	0.0971
Transferred from an outside hospital	84 (95)	29 (85)	0.0045
Antibiotics before diagnosis	19 (22)	8 (24)	0.8131

∧ *21 patients missing cell count; 24 patients missing percentage polymorphonucleocytes (%PMN); and positive erythema status.*

*Results shown are in median [interquartile range (IQR)] or n (%).*

*P-value from Kruskal–Wallis test for the continuous variables and Fisher's exact test for the categorical variables*.

The patients with true septic knee arthritis and presumed septic knee arthritis differed significantly with respect to the CRP level (median CRP level of 5.3 mg/dl for true septic knee and 3.3 mg/dl for presumed septic knee); all other variables did not differ significantly among the groups.

Of the 88 patients with septic knee arthritis (true and presumed), two patients were missing temperature and three were missing ESR. Therefore, a total of 83 patients with septic knee arthritis and 34 patients with aseptic knee effusion were included in the initial regression modeling. In a multivariable regression model that included Kocher's original four criteria and Caird's modification (ESR > 40 mm/h, temperature > 38.5°C, weight bearing status [weight bearing, non-weight bearing, or unknown], serum WBC count >12,000 cells/mm^3^, and CRP > 2 mg/dl), none of the criteria reached statistical significance.

**Figure 2:**
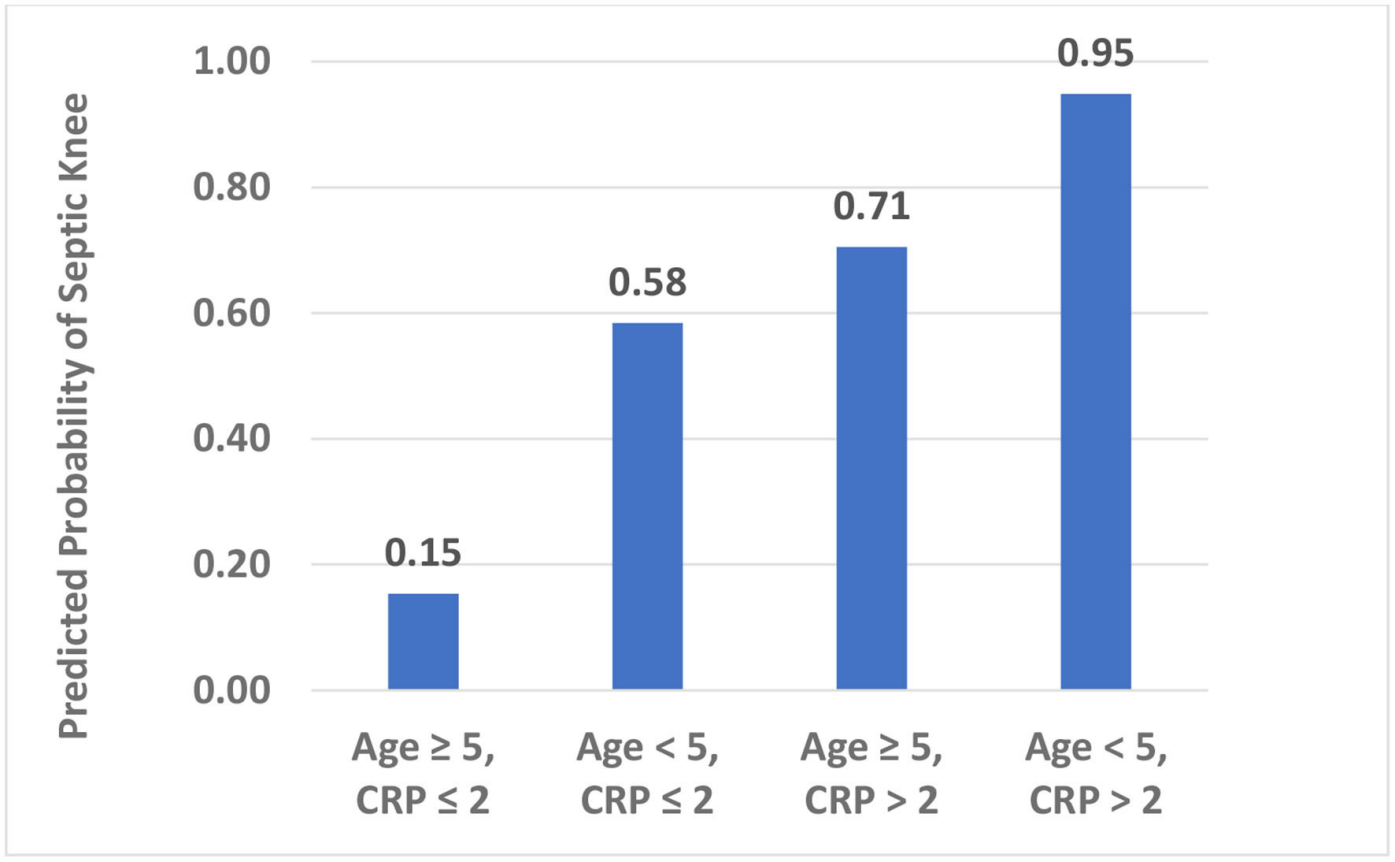
Predicted probability of a septic knee by age (years) and CRP (mg/dl).

**Figure 3:**
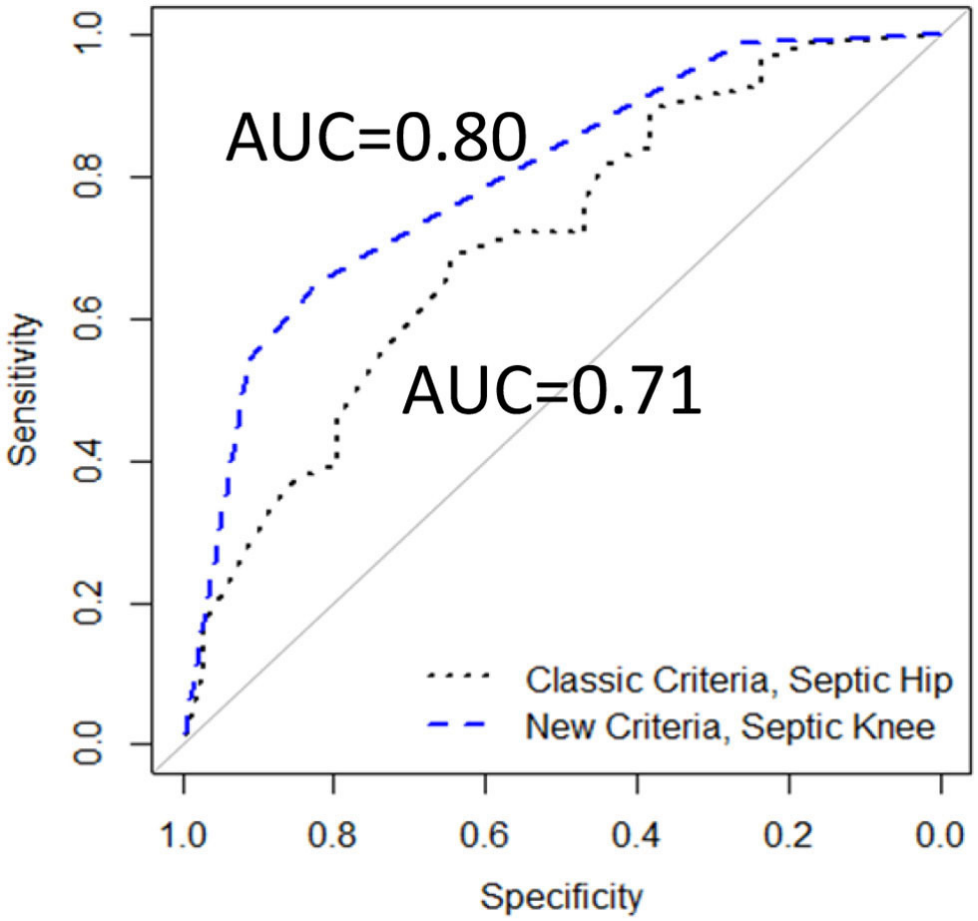
The receiver-operator curves (ROCs) comparing Classic Criteria model to a New Criteria model, including age and CRP (117 patients), AUC, area under the curve.

We examined a model using a modification of the hip criteria plus age selected *a priori*: CRP > 2 mg/dl, temperature > 38.5°C, serum WBC > 12,000 cells/mm^3^, weight bearing status, and age <5 years. In this model, the younger age was significantly associated with the higher risk of having septic arthritis of the knee, while the CRP, temperature, serum WBC count, and weight bearing status were not significantly associated with the chances of having septic arthritis of the knee. After applying the backward elimination criteria, our knee model contained age <5 years and CRP > 2 mg/dl (Table 2). When calculating the risk extremes using the model-based estimates, a patient presenting with age ≥5 years and CRP ≤ 2 mg/dl would have the lowest risk probability of 0.15 for having septic arthritis of the knee. In contrast, a patient presenting with age <5 years and CRP > 2 mg/dl would have the highest risk probability of 0.95 for having septic arthritis of the knee (Figure 2). Comparing the ROCs for our proposed criteria for the septic knee to that generated by the classic hip criteria revealed that the AUC were not significantly different, with the proposed septic knee criteria AUC = 0.80 vs. the classic hip criteria AUC = 0.71, *p* = 0.12 (Figure 3).

**Table 2:** The logistic regression results, septic knee (n = 83) vs. aseptic knee (n = 34), a new septic knee model with (A) all pre-specified variables and (B) reduced set of variables.

**Variable**	**A.Full model**		**B.Reduced model**	
	**Odds ratio** **(95% CI)**	***P*** **-value**	**Odds ratio** **(95% CI)**	***P*** **-value**
Age < 5	11.82 (3.90, 45.56)	<0.001	13.16 (4.50, 49.41)	<0.001
CRP > 2	6.22 (1.67, 27.55)	0.01	7.73 (2.33, 31.22)	0.002
TEMP > 38.5	1.26 (0.41, 4.02)	0.69	–	–
WBC > 12k	1.54 (0.59, 4.11)	0.38	–	–
WB No vs. Yes	1.68 (0.57, 4.87)	0.34	–	–
WB unknown vs. Yes	1.11 (0.16, 8.37)	0.91	–	–

The mean cell counts and differential for 51 patients with a culture positive septic arthritis of the knee or a positive blood culture with synovial cell counts over 50,000 cells/mm^3^ were 94,500 cells/mm^3^ and 87% polymorphonucleocytes. Of note, nine of the 51 patients with culture positive septic arthritis of the knee in our study had synovial fluid white blood cell counts *under* 50,000 cells/mm^3^ (mean 22,000 cells/mm^3^, range 839–40,250 cells/mm^3^) but they did tend to have elevated %PMN (mean 86% polymorphonucleocytes, range 66–98%).

**Figure 4:**
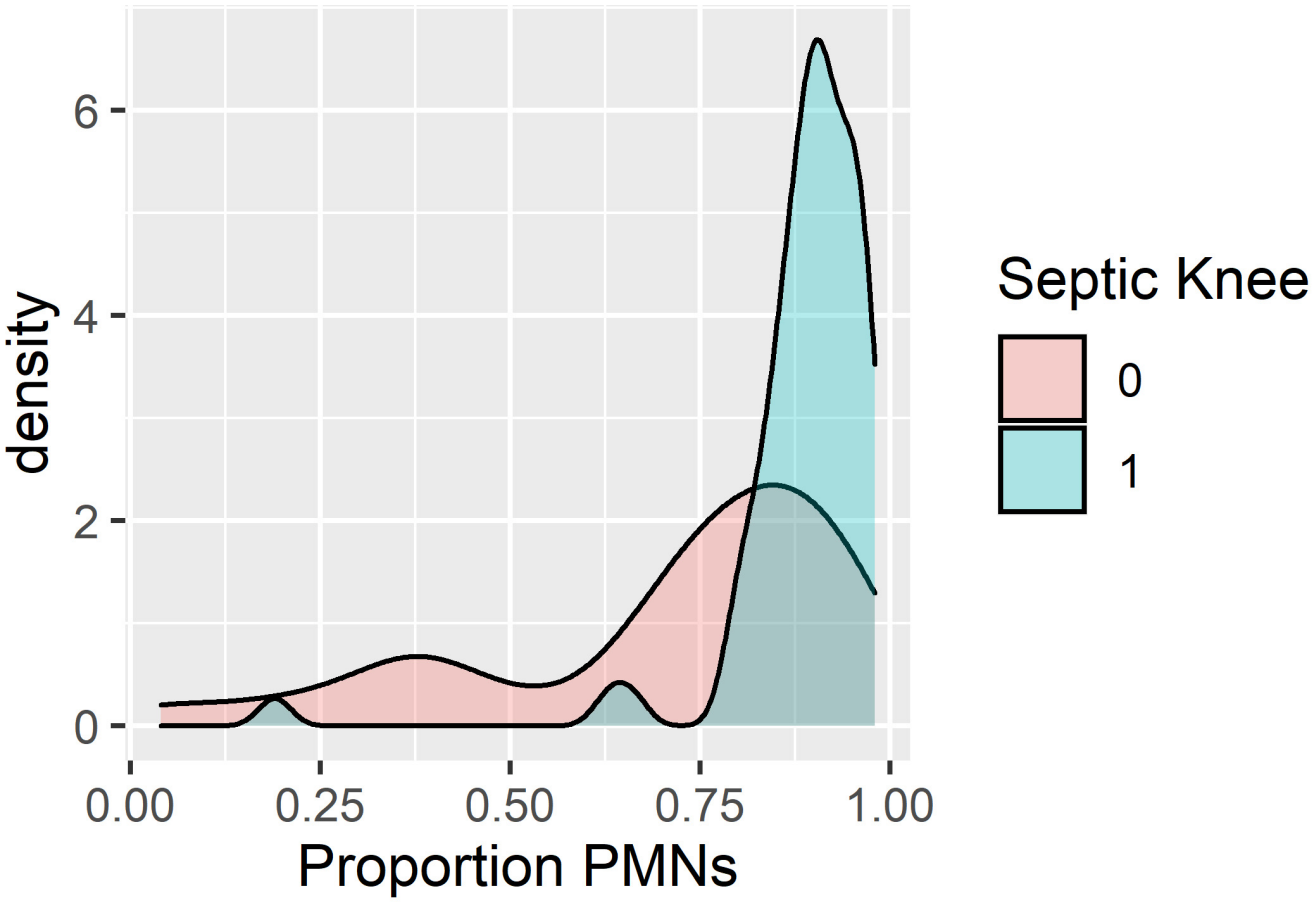
Density plot of patients by percentage polymorphonucleocytes (%PMN) for known septic knee vs. aseptic effusion.

**Figure 5:**
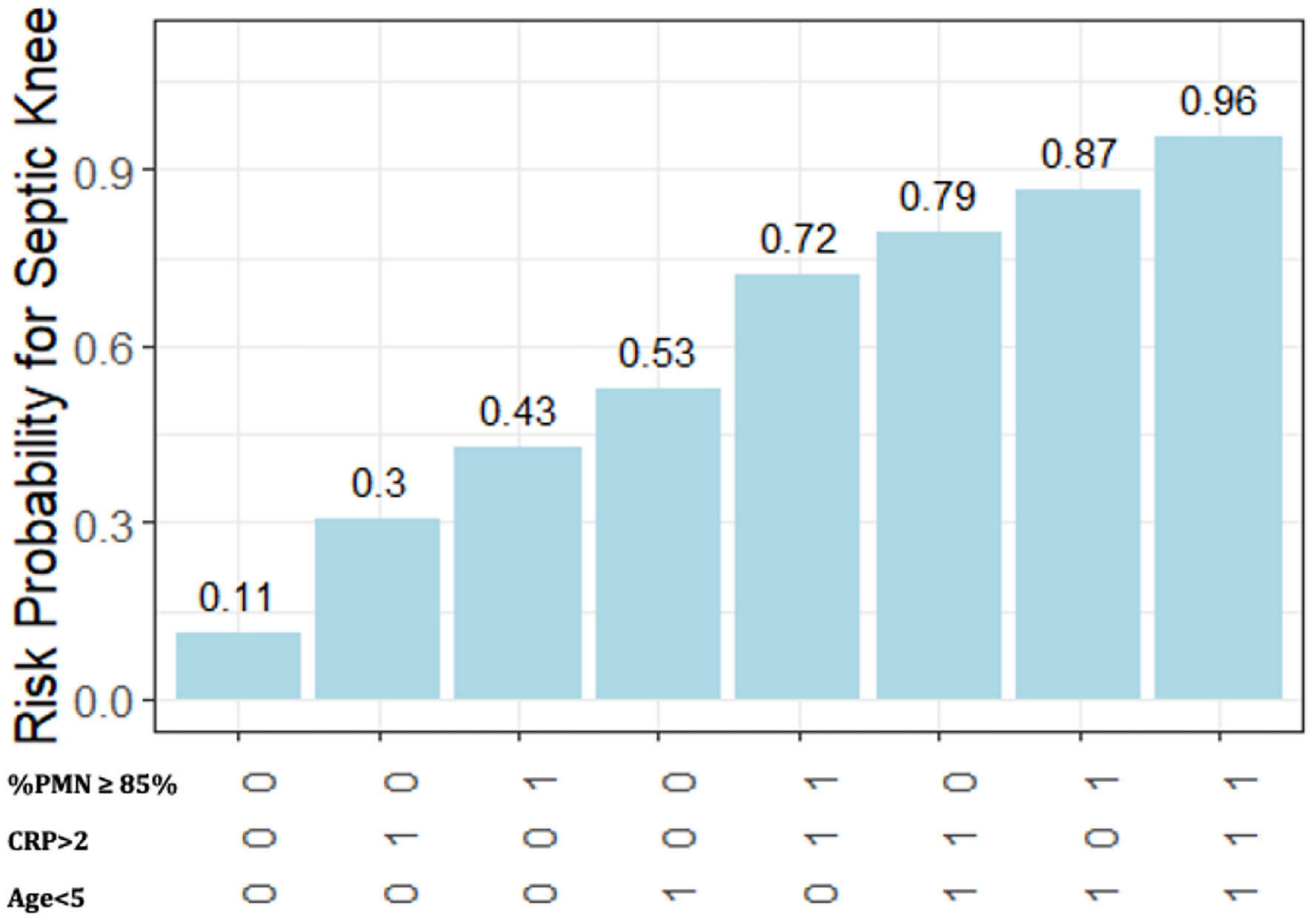
Predicted probability for septic knee based on the predictor variable combinations.

Given the meager improvement of the knee model over the AUC of the classic hip model, we added %PMN to the predictive model in a secondary analysis. In addition, 24 patients were missing %PMN and were excluded. Reviewing the density plots of %PMN for those with septic arthritis and aseptic effusion led to the assignment of a cut-off at 85% polymorphonucleocytes for the transition to “likely infected” (Figure 4). After revisiting the previously examined variables and applying the same backward elimination criteria, three variables remained: age, CRP, and %PMN (Table 3). The highest risk probability of septic arthritis of the knee was 96% for the patients less than the age of 5 years, with CRP > 2, and %PMN ≥ 85%, and the lowest risk probability of septic arthritis was 11% when patients were ≥5 years of age, with CRP ≤ 2, and %PMN <85% (Figure 5).

**Table 3:** The logistic regression results, septic knee (*n* = 64) vs. aseptic knee (*n* = 29), new septic knee model with (A) all the pre-specified variables and (B) reduced set of variables (subset with non-missing %PMNs).

**Variable**	**A.Full model**		**B.Reduced model**	
	**Odds ratio (95% CI)**	***P*** **-value**	**Odds ratio (95% CI)**	***P*** **-value**
Age < 5	8.18 (2.37, 35.40)	0.002	8.70 (2.69, 35.28)	0.001
CRP > 2	3.41 (0.72, 18.45)	0.13	3.40 (0.82, 15.95)	0.10
TEMP > 38.5	1.06 (0.28, 4.11)	0.94	–	–
WBC > 12k	1.40 (0.45, 4.41)	0.56	–	–
WB No vs. Yes	1.32 (0.37, 4.67)	0.66	–	–
WB unknown vs. Yes	0.43 (0.05, 3.94)	0.45	–	–
PMNs ≥ 85%	6.08 (1.97, 20.37)	0.002	5.85 (1.95, 18.84)	0.002

**Figure 6:**
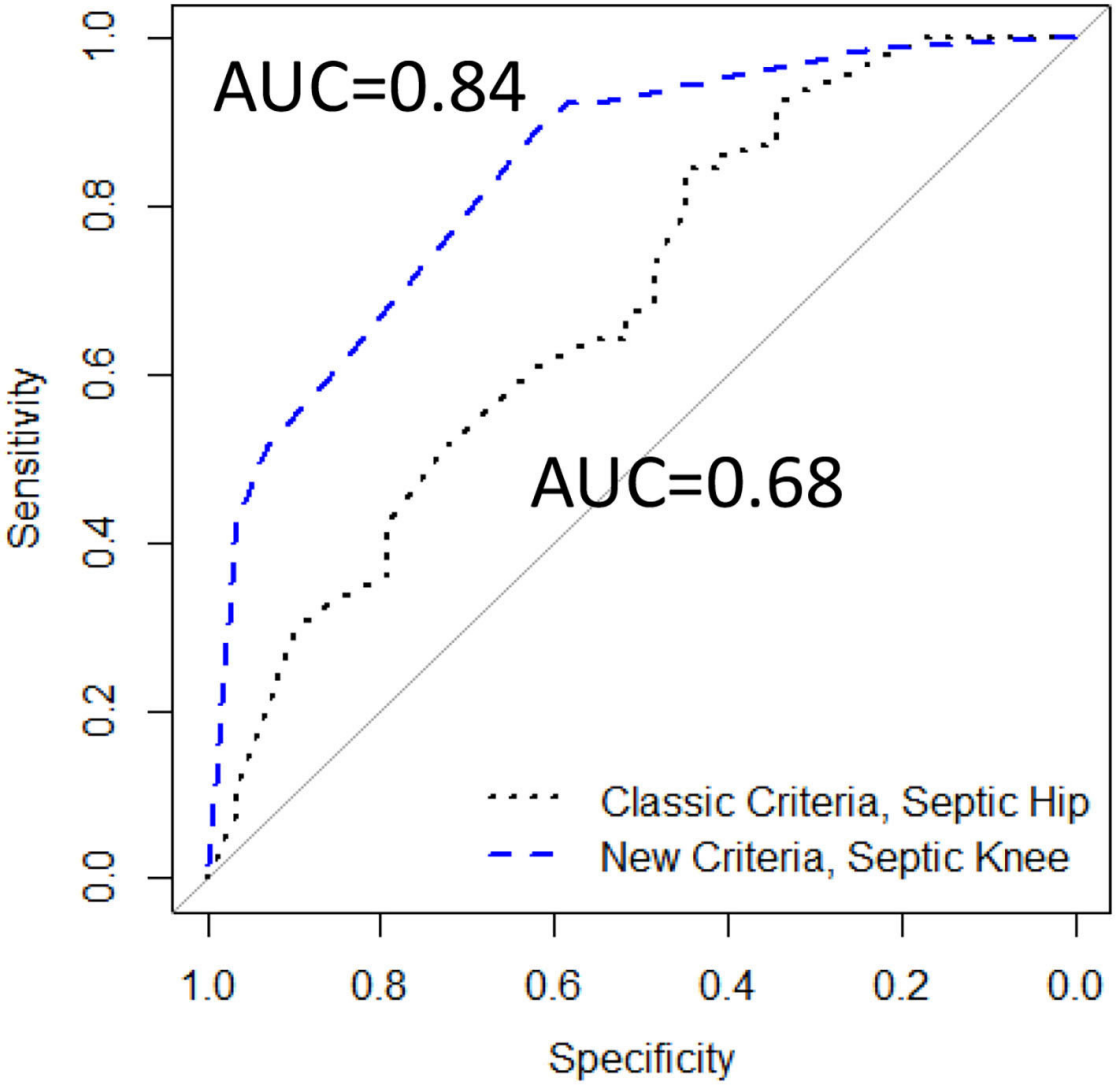
The ROCs comparing Classic Criteria model (117 patients) to a *modified* New Criteria model containing age, CRP, *and* %PMN (93 patients), AUC, area under the curve.

Comparing the ROCs again revealed that the AUC for this new septic knee model was 0.84, which was significantly different from that of the classic septic hip criteria of 0.68 (*p* = 0.02) (Figure 6).

One patient in this study was formally diagnosed with streptococcal pharyngitis and one with Kawasaki's disease, though additional patients may have been included who were not formally diagnosed. Six patients with known juvenile idiopathic arthritis were included; three of them were having aseptic effusions, two had presumed septic arthritis (one with 51,000 WBC on aspiration and 93% polymorphonucleocytes; one with 64,000 WBC on aspiration and 88% polymorphonucleocytes), and one was diagnosed with culture positive septic arthritis. Of note, our algorithm did not change whether we included these six patients or not. Lyme disease is not endemic to the area of this study, though one patient had positive Lyme titers and grew Strep viridans from their blood cultures.

## Discussion

This study examines the predictors for septic arthritis of the knee in isolation. While the knee joint does not carry a specific non-toxic diagnosis akin to transient synovitis of the hip, non-toxic effusions leading to the painfully swollen knees do exist and must be distinguished from septic arthritis. In this study, they were termed “aseptic knee effusion,” and may have included Reiter's syndrome, post-streptococcal reactive arthritis, juvenile idiopathic arthritis, and rheumatic fever.

In Kocher's validation study of septic arthritis of the hip, a child with four out of four criteria (elevated ESR, serum WBC count, temperature, and non-weight bearing status) had a predicted probability of 93% of having septic hip arthritis, which was diminished slightly from the original study in which the probability was 99.8% (4, 5). However, Luhman et al. noted that when all the four Kocher criteria were present in a population of patients in a Midwestern urban area, the risk of septic arthritis of the hip was only 71% (13). Levine et al. later showed that CRP was a better independent predictor of pediatric septic arthritis than ESR and an especially good negative predictor for the disease (14). Of note, their study included hip (24%), knee (49%), and other joint (27%) septic arthritis, and was performed in a Lyme endemic area, which potentially diminished the positive predictive value of CRP. Caird then updated Kocher's criteria to include CRP and found a 97.5% predictive probability of septic hip arthritis, if all the five criteria were present (6). Singhal et al. worked to simplify these criteria by showing that CRP and weight bearing status, when taken together as the only two predictive criteria, offered a 74% predicted probability of septic arthritis (15). In that study, however, the patients in the transient synovitis group were largely treated without aspiration of the hip and therefore differed from the patients in the previous studies. Baldwin et al. suggested criteria that may distinguish pyogenic septic arthritis of the knee from Lyme monoarticular arthritis, namely a short motion arc, CRP >4.0 mg/dl, reported history of fever, and age younger than 2 years (16). In their study, the presence of these four criteria suggested a 100% probability of septic arthritis. However, as Baldwin noted, their clinical prediction algorithm may not be applicable in the non-endemic areas with a lower prevalence of Lyme disease (16). Joshy compared the pediatric patients with septic arthritis of the hip and knee and found that the patients with septic knee arthritis tended to be younger than the patients with septic hip arthritis (mean age 2.5 vs. 4.8 years) and were less likely to have positive synovial fluid culture (19 vs. 40%) (7). Of the seven patients in their study with culture-positive septic knee arthritis, only three presented with three or more Kocher criteria, whereas all the 12 patients with culture-positive septic hip arthritis presented with three or more Kocher criteria. Obey et al. recently showed that the sensitivity of having three or more Kocher criteria was only 48.5% in children with septic knee arthritis, meaning over half of the children with septic arthritis of the knee would be missed even if they had three out of four Kocher criteria. This improved to 71.6% if CRP was one of the three criteria (9).

Obana et al. reviewed the patients with joint effusions not limited to the hip or knee, identifying the value of examining %PMN in determining the likelihood of septic arthritis. In their population of 166 patients who underwent 172 joint aspirations, they found one of the most reliable factors predictive of septic arthritis to be %PMN from the aspirate, where the mean %PMN for the true and presumed septic arthritis patients to be 81%, and only ~60% for the un-infected patients (10). Avoiding a joint aspiration, when possible, for the patients with an irritable hip makes sense, as it may mean avoiding a general anesthetic. However, aspiration of a knee may merely require sedation, and the knee joint aspiration and sedation are the two procedures easily performed in a pediatric emergency department.

Our algorithm suggests that the children under 5 years of age with an elevated CRP level presenting with a painful swollen knee have a 95% predicted probability of having septic arthritis of the knee. This knowledge should help dictate the future care of these patients. For those patients of age ≥5 years and CRP ≤ 2 mg/dl, the likelihood for septic arthritis remains concerning at 15%. Adding a knee aspiration would be beneficial, as synovial fluid white blood cell counts >50,000 cells/mm^3^ would suggest that such patients would likely benefit from irrigation and debridement of the joints, though of note, 11 of the 54 patients with culture positive septic arthritis of the knee in our study had synovial fluid white blood cell counts *under* 50,000 cells/mm^3^. Obtaining aspirate differentials would be useful, as %PMN <85% would decrease the likelihood of septic arthritis to 11%, and enabling a culture of synovial fluid. Finding %PMN to be ≥85% would increase the risk of septic arthritis of the knee to 43% in this group of patients.

### Limitations

As Kocher found, our patients with presumed septic arthritis had symptoms and signs similar to those with true septic arthritis, including high joint aspirate white blood-cell count counts. Whether this presumed septic arthritis actually represents bacterial arthritis with organisms that are difficult to grow on culture, viral arthritis, arthritis from atypical organisms, inflammatory or juvenile idiopathic arthritis, trauma, periarticular osteomyelitis, or an autoimmune process is impossible to determine.

This study neither address the *treatment* nor the *outcomes following* pediatric septic knee arthritis but instead focuses on the clinically relevant *predictors* of the diagnosis. This diagnostic algorithm will have to be validated in an independent dataset. While the AUC of this model was 0.84, indicating a good diagnostic performance, the model would likely perform differently when applied to a new set of patients. Our population of patients included all those with a painful swollen knee that underwent a knee joint aspiration, likely skewing the population of all those presenting with painfully swollen knees toward the sicker, more severely involved patients. Whether this algorithm would apply to all children presenting with a painful swollen knee remains to be seen. In addition, this is a *proposed* clinical prediction algorithm that will need to be validated in additional population. As this was a retrospective study with the goal of identifying indicators of septic arthritis vs. non-infectious effusions, we included all the qualifying patients during the time-period of interest. We did not do a power calculation; hence, our study may be underpowered to identify the statistically significant factors.

Though the use of a laboratory measure, such as CRP in both the prediction algorithm and as a factor in the determination of a final diagnosis by Infectious Disease and Orthopedic Services may be seen as a limitation, the final diagnoses were made after careful consideration of *trends* in the patient symptoms, clinical signs, and laboratory values over time, in the face of various treatment modalities, such as observation with or without anti-inflammatory medications, antibiotic treatment, and irrigation of the knee joint.

An additional limitation of the study is the ever-increasing regions where Lyme disease is endemic, making the applicability of this prediction tool less useful over time. However, in non-Lyme endemic areas, were a patient to have a likely exposure to Lyme disease and a concern for septic arthritis, serum sampling for the C6 enzyme immunoassay test should be considered, as a positive or equivocal test result had a high sensitivity (100%) and specificity (94%) for Lyme disease in a multicenter study by Nigrovic et al. (17), and such a result may prevent the unnecessary arthrocentesis.

In conclusion, this study examines pediatric patients with painfully swollen knees to validate the classic prediction algorithm previously described for hips. We found that the children with septic knee arthritis do not routinely follow the clinical criteria for a septic hip, and therefore, that model may not reliably distinguish the septic knee arthritis from aseptic knee effusion. Second, this study offers a different prediction algorithm for the pediatric patient with a painful swollen knee treated in non-Lyme endemic areas that includes age and CRP level, that when taken together can help predict the likelihood of septic knee arthritis. A patient presenting with a painful swollen knee and age younger than 5 years and CRP >2.0 mg/dl would have greater than a 95% likelihood of having septic arthritis of the knee. Conversely, if the patient has a painful swollen knee but neither of these two factors, the likelihood of septic arthritis of the knee would be 15%; a knee joint aspirate with %PMN <85% would lower the risk to 11%, as well as providing synovial fluid for culture. This model should guide the practitioners who are evaluating a pediatric patient with a painful swollen knee.

## Data Availability Statement

The raw data supporting the conclusions of this article will be made available by the authors, without undue reservation.

## Ethics Statement

The studies involving human participants were reviewed and approved by Seattle Children's Institutional Review Board, STUDY00000673. Written informed consent from the participants' legal guardian/next of kin was not required to participate in this study in accordance with the national legislation and the institutional requirements.

## Author Contributions

CT planned this study, performed much of the data collection and analysis, wrote the initial draft, and approved the final submission. GS oversaw all elements of the project, reviewed all data, oversaw the data analyses, and wrote the final text of the paper. VB contributed to the study design, oversaw and participated in data collection, and approved the final submission. AF aided in the study design, performed the statistical analyses, and approved the final submission. CS aided in data collection, data analysis, and approved the final submission. All authors contributed to the article and approved the submitted version.

## Conflict of Interest

The authors declare that the research was conducted in the absence of any commercial or financial relationships that could be construed as a potential conflict of interest.

## Publisher's Note

All claims expressed in this article are solely those of the authors and do not necessarily represent those of their affiliated organizations, or those of the publisher, the editors and the reviewers. Any product that may be evaluated in this article, or claim that may be made by its manufacturer, is not guaranteed or endorsed by the publisher.
